# Defining core signaling pathways for supporting *in vitro* maintenance of pig extraembryonic endoderm (XEN) cells

**DOI:** 10.1530/REP-24-0393

**Published:** 2025-03-11

**Authors:** Jinsol Jeong, Dong-Kyung Lee, Kwang-Hwan Choi, Dong-Wook Kim, Seokjong Lee, Jong-Nam Oh, Yelim Ahn, Chang-Kyu Lee

**Affiliations:** ^1^ Department of Agricultural Biotechnology, Animal Biotechnology Major, and Research Institute of Agriculture and Life Science, Seoul National University, Seoul, Korea; ^2^ Research and Development Center, Space F corporation, Hwasung, Gyeonggi-do, Korea; ^3^ Institute of Green Bio Science and Technology, Seoul National University, Pyeong Chang, Kangwon-do, Korea

**Keywords:** XEN, primitive endoderm, pig, serum-free, signaling pathway, stem cells

## Abstract

Extraembryonic endoderm (XEN) cells can be derived from blastocyst primitive endoderm (PrE), becoming a useful tool for studying mammalian development, including early lineage segregation and embryo patterning. Establishment of stem cells representing the respective lineages in blastocysts has been robustly attempted in domestic animals, especially pigs, to reconstitute embryogenesis *in vitro* for comparative studies. Therefore, we developed a serum-free culture system for pig XEN cells by dissecting the signals governing the core gene network of the PrE lineage. The FGF, LIF and WNT signaling pathways and B27 supplements are essential for maintaining a rapid proliferation rate in pig XEN cells. These cells recapitulated the molecular features and differentiation capacity of the PrE lineage. Especially, the XEN cells incorporated into normal development, retaining cellular identity and contributing to the PrE lineage when injected into *in vitro*-produced porcine blastocysts. In addition, species-specific characteristics of pigs were observed, including the involvement of lipid metabolism and NANOG/GATA co-expression in XEN cells. Taken together, our findings can contribute to the expansion of the understanding of developmental biology and its biomedical applications by enabling reproducible and homogeneous porcine XEN cell culture.

## Introduction

The separation of the inner cell mass (ICM) and trophoblast in early blastocysts (BLs) is the first lineage segregation, followed by the commitment of ICM cells to epiblast (Epi) or primitive endoderm (PrE)/hypoblast fate (Yamanaka *et al.* 2010). Three distinct stem cell types, embryonic stem cells (ESCs), extraembryonic endoderm (XEN) cells and trophoblast stem cells (TSCs), are derived from the Epi, PrE and trophectoderm (TE), respectively. Of these, XEN cells represent the PrE lineage, which contributes to the parietal endoderm and visceral endoderm of the yolk sac as extraembryonic endoderm *in vivo* (Ralston 2018). The establishment of XEN cells has been studied primarily in mice, followed by rats, humans and pigs (Kunath *et al.* 2005, Seguin *et al.* 2008, Debeb *et al.* 2009, Park *et al.* 2021). Although the reported XEN cell lines showed species-specific differences such as signaling pathways for maintenance, they were generally capable of recapitulating the molecular signature and biological functions of the PrE lineage. The PrE lineage differentiates into the extraembryonic endoderm *in vivo*, which influences surrounding fetal tissues for cell fate specification at the correct spatial and temporal developmental window, such as lineage segregation, axis patterning and germ cell differentiation (Ralston 2018). Accordingly, XEN cells enable in-depth studies of embryogenesis as a state of self-renewing stem cells since they can serve as an equivalent of the *in vivo* PrE lineage and its derivative, extraembryonic tissue.

The first reported XEN cells were reproducibly derived from mouse BLs and expanded without senescence *in vitro* (Kunath *et al.* 2005). Self-renewing XEN cells were characterized by the expression of PrE lineage-associated markers and differentiation ability toward the extraembryonic endoderm lineage in chimeras. Thereafter, alternative methods to derive XEN cells, such as separation from TSCs in seeded post-implantation embryos (Lin *et al.* 2016), overexpression of the PrE marker *Gata4/6* or directed differentiation using signaling molecules in ESCs (Fujikura *et al.* 2002, Niakan *et al.* 2013), or reprogramming of somatic cells undergoing a XEN-like intermediate (Zhao *et al.* 2015), have been discovered. These studies suggest that PrE lineage-representing cells distinct from ESC and TSC lines can be obtained *in vitro*, which provides an opportunity to reveal the mechanism underlying PrE development. In fact, XEN cells have been recently applied in 3D embryo modeling, uncovering the embryo developmental process, including XEN cell-derived PrE lineage specification (Zhang *et al.* 2019, Ohinata *et al.* 2022). In these developmental mechanism studies, reliable results can be obtained through culture systems that are capable of providing reproducible and homogeneous stem cells. However, in most studies, culture systems of XEN cells were based on the maintenance of ESCs or TSCs rather than XEN cells and used fetal bovine serum (FBS)-containing media, accompanied by serum-derived defects, such as batch-to-batch variation and unknown factors (Niakan *et al.* 2013). In this regard, a XEN cell-specific and serum-free medium is a prerequisite for a consistent culture of them.

In pigs, XEN cells have been misunderstood and studied as epiblast stem cells, previously referred to as ‘embryonic stem-like cells’ (Park *et al.* 2013, 2021), because the overgrowth of PrE cells compared to Epi cells occurs during the establishment of epiblast-derived stem cells as well as that of XEN cells (Park *et al.* 2021). With the establishment of authentic porcine ESCs, recent stem cell research, including XEN cells in domestic animals such as pigs, has been robustly studied for biomedical and agricultural purposes (Choi *et al.* 2019). Since porcine embryo development is distinct from that of mice and humans in terms of an extended pre-implantation period, the signaling mechanism involved in the maintenance and specification of BL-derived PrE lineage in pigs can be distinct from that in other species. Several studies on pig XEN cells have been conducted under serum-free conditions supplemented with signaling molecules (Shen *et al.* 2019, Li *et al.* 2020), but these culture conditions compromised the self-renewal ability of XEN cells when compared to FBS-containing media (Park *et al.* 2021), possibly because of inappropriate extrinsic cues. Therefore, in this study, we aim to develop an optimized serum-free culture system by identifying porcine XEN-specific signaling pathways, where XEN cells can function as the PrE lineage and their derivatives *in vitro* and *in vivo*.

## Materials and methods

### Ethics declarations

The care and experimental use of LYD pigs (*Sus scrofa*) and mice (*Mus musculus*) were approved by the Institutional Animal Care and Use Committee of Seoul National University (approval no. SNU-140328-2 and SNU-191025-4-4). Pig ovaries were donated from a local slaughterhouse (Anyang, Korea). Pregnant ICR mice were purchased from Samtako Bio (Republic of Korea). All methods were performed according to the guidelines and regulations of the Institutional Animal Care and Use Committee of Seoul National University.

### Derivation and culture of pig extraembryonic endoderm (XEN) cells

Porcine embryos were produced as in the previous study (Choi *et al.* 2019). Hatched BLs were seeded on mitotically inactivated mouse embryonic fibroblasts with a medium composed of Dulbecco’s modified Eagle’s medium (DMEM)/F12, 1× GlutaMax, 0.1 mM β-mercaptoethanol, 1× antibiotic–antimycotic, 15% (v/v) KSR (all from Gibco, USA), 10 ng/mL human recombinant LIF (Millipore, USA) and 10 ng/mL FGF2 (R&D Systems, USA), as previously reported (Oh *et al.* 2022). Approximately 14 days after seeding, primary colonies of the PrE cell type were dissociated using pulled glass pipettes and transferred onto new feeder cells for subculture.

To culture pig XEN cells derived from PrE cell type colonies, several signaling molecules and supplements were tested in a medium composed of DMEM/F12, 1× GlutaMax, 0.1 mM β-mercaptoethanol, 1× antibiotic–antimycotic and 5% (v/v) KSR (all from Gibco). The tested factors were 10 ng/mL FGF2 (R&D Systems), 10 ng/mL human recombinant LIF (Millipore), 5 ng/mL ActA (R&D Systems), 1.5 μM GSK3B inhibitor CHIR99021 (Cayman Chemical, USA) and 1× B27 supplements (Gibco). After optimization of culture conditions, pig XEN cells were cultured in XEN cell culture medium (DMEM/F12, 1× GlutaMAX, 0.1 mM β-mercaptoethanol, 1× antibiotic–antimycotic, 5% (v/v) KSR (all from Gibco), 1× B27 supplements (Gibco), 10 ng/mL FGF2 (R&D Systems), 10 ng/mL hrLIF (Millipore) and 1.5 μM CHIR99021 (Cayman Chemical)). At 24 h before subculturing, the cells were cultured with XEN cell culture medium containing 10 μM Y-27632 (Santa Cruz Biotechnology, USA). The expanded colonies were dissociated into small clumps using TrypLE Express (Gibco). These clumps were transferred onto new feeder cells with 1:10 ratio every 3–4 days and cultured in XEN cell culture medium containing 10 μM Y-27632 for 24 h. Attached clumps were then cultured in XEN cell culture medium lacking Y-27632. The medium was changed every 24 h, and pig XEN cells were cultured under humidified conditions in an atmosphere containing 5% CO_2_ and 5% O_2_ at 38°C. The mycoplasma test was performed using the e-Myco™ plus Mycoplasma PCR Detection Kit (iNtRON Biotechnology, Inc., Korea) according to the manufacturer’s protocol.

### Porcine ESCs (PESCs) culture

Porcine ESCs (PESCs) were cultured on feeder cells as previously reported (Choi *et al.* 2019). Briefly, ESC medium supplemented with 20 ng/mL FGF2 (R&D Systems), 5 ng/mL ActA (R&D Systems), 1.5 μM CHIR99021 (Cayman) and 2.5 μM IWR-1 (Sigma-Aldrich, USA) was changed every 24 h. Cells were cultured under humidified conditions in an atmosphere containing 5% CO_2_ at 37°C.

### XEN-derived spheroid formation for inducing differentiation toward PrE lineages

To evaluate *in vitro* differentiation ability, XEN-derived spheroids were generated from pig XEN cells. Cultured cells were dissociated into small clumps using TrypLE Express and passaged using XEN cell culture medium containing 10 μM Y-27632 into ultra-low-attachment plates (Sigma-Aldrich). After 24 h, the cells were cultured in DMEM (Welgene, Korea) supplemented with 10% (v/v) FBS (collected and processed in the United States; GeneDepot, USA), 1× GlutaMax, 0.1 mM β-mercaptoethanol, 1× antibiotic/antimycotic and 10 μM Y-27632 (day 1 only) for 5 days. During suspension culture, the cells aggregated and formed XEN-derived spheroids.

### Generation of EGFP-XEN cells

The pHIV-EGFP plasmid (Addgene, plasmid #21373) was used to generate EGFP-tagged XEN cells. The linearized plasmids were transfected into XEN cells using the Neon^®^ Transfection System (Thermo Fisher Scientific, USA) according to the manufacturer’s instructions. Subsequently, EGFP-positive XEN (EGFP-XEN) cells were separated through fluorescence-activated cell sorting (FACS) using a FACSAria II (BD Biosciences, USA) for further analysis.

### Blastocyst injection

EGFP-XEN cells, cultured with 10 μM Y-27632 for 24 h, were separated from feeder cells through manual colony picking using pulled glass pipettes. Then, they were dissociated into single cells using TrypLE Express and resuspended with XEN cell culture medium containing 10 μM Y-27632. Five to eight cells were injected into pig embryos 5 days after parthenogenetic activation, which were produced as previously reported (Choi *et al.* 2019). The injected embryos were cultured for another 48 h, followed by fixation for further analysis.

### Alkaline phosphatase (AP) staining

After washing with Dulbecco’s phosphate-buffered saline (DPBS; Welgene), fixed cells were stained with a solution containing nitro blue tetrazolium chloride (NBT) and 5-bromo-4-chloro-3-indolyl phosphate toluidine salt (BCIP) stock solution (Roche, Switzerland) in a buffer solution for 30 min at room temperature. The cells were then examined under an inverted microscope.

### Oil Red O staining

After washing with DPBS (Welgene), fixed cells were washed with 60% (v/v) isopropyl alcohol (Sigma-Aldrich) for 5 min at RT and stained with a filtered solution containing Oil Red O (Sigma-Aldrich) for 20 min at RT. After washing with UltraPure Water (Welgene), images of stained cells were captured under an inverted microscope.

### Immunocytochemistry (ICC) analysis

The samples were fixed in 4% (w/v) paraformaldehyde for immunostaining. After washing with DPBS, the fixed cells and embryos were treated with 0.2% Triton X-100 (Sigma-Aldrich) for 2 h at 4°C and 1% Triton X-100 for 1 h at RT, respectively. After washing, the samples were treated with 10% (v/v) goat serum or 1% (v/v) bovine serum albumin in DPBS for 2 h. Serum-treated samples were incubated with primary antibodies for 24 h at 4°C. The primary antibodies used were rabbit anti-SOX2 (1:200, Millipore; AB5603), rabbit anti-NANOG (1:200, PeproTech, USA; 500-P236), chicken anti-OCT4 (1:100, Abcam, UK; ab134218), goat anti-GATA6 (15 μg/mL; R&D Systems; AF1700), goat anti-SOX17 (1:200, R&D Systems; AF1924), rabbit anti-GATA4 (1:200, Abcam; ab84593) and chicken anti-GFP (1:100, Invitrogen, USA; A10262). After washing, the samples were incubated for 24 h at 4°C or 2 h at RT with an Alexa Fluor-conjugated secondary antibody. After washing, cells were stained with Hoechst 33342 (Molecular Probes, USA), and embryos were mounted on slide glass with ProLong Gold with DAPI (Invitrogen). Images of stained cells were captured using a CELENA X high-content imaging system (Logos Biosystems, Korea).

### Quantitative real-time polymerase chain reaction (qPCR)

Total RNA was extracted from the cells using TRIzol^Ⓡ^ Reagent (Invitrogen) and purified by treating with RNase-free DNase I (Thermo Fisher Scientific) to remove genomic DNA, according to the manufacturer’s instructions. RNA with an A260/280 ratio of 2.0 or higher, confirming its purity via spectrophotometer, was used. cDNA was synthesized using a High-Capacity RNA-to-cDNA Kit (Applied Biosystems, USA). The derived cDNA samples were amplified with PowerSYBR^Ⓡ^ Green PCR Master Mix (Applied Biosystems) containing each primer set listed in Supplementary Table S1 (see section on Supplementary materials given at the end of the article). Primers validated using the following method were used: PCR products generated with a PCR Master Mix solution (iNtRON) in a Thermal Cycler (Thermo Fisher Scientific) were size-verified through gel electrophoresis, and the purified amplicons, obtained using the MEGAquick-spin™ Plus Total Fragment DNA Purification Kit (iNtRON), were sequenced. Amplification and detection were conducted using an ABI 7300 Real-Time PCR system (Applied Biosystems). The relative expression levels were calculated by normalizing the threshold cycle (Ct) values of each gene to that of *GAPDH* using the ΔCt method (Livak & Schmittgen 2001).

### Statistical analysis

The data from the qPCR analyses are presented as the mean ± standard error of the mean (SEM) and were analyzed using Prism 6 software (GraphPad Software, USA). The significance of differences was determined by *t*-test or one-way analysis of variance (ANOVA), followed by Fisher’s least significant difference (LSD) test. Differences were considered statistically significant at *P* < 0.05.

### Karyotyping

Karyotyping of cells using standard G-banding chromosomes and cytogenetic analysis were performed at GenDix Laboratories (www.gendix.com; Korea).

## Results

### Optimization of serum-free culture conditions for pig XEN cells from *in vitro*-produced BLs

**Figure 1:**
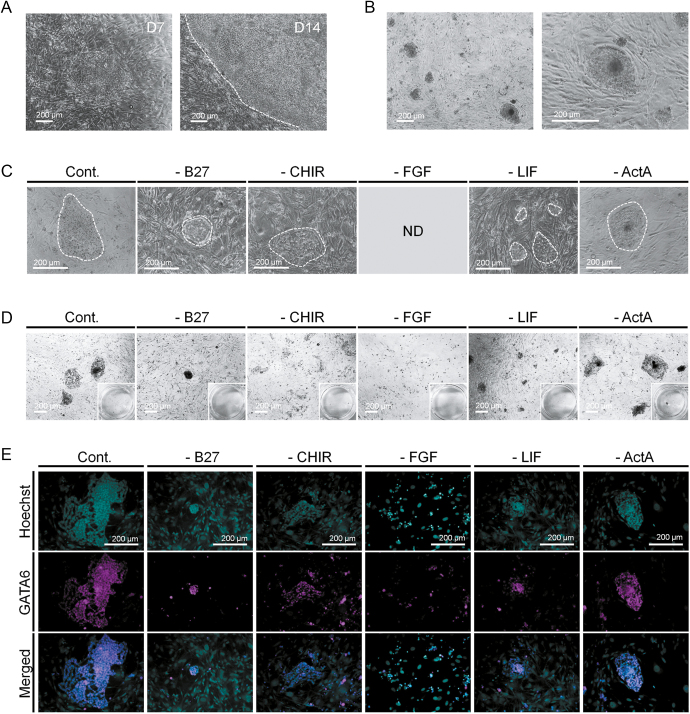
Screening of culture conditions for pig XEN cells. (A) XEN cells derivation from seeded BLs. (B) Morphology of pig XEN cell during maintenance. (C, D and E) Effect of signaling molecule or supplement withdrawal on XEN cells. (C) Cell images. ND: not detected. (D) AP-stained images. (E) Immunostaining for XEN cell markers.

To develop a serum-free medium, we selected four signaling molecules, including CHIR99021 (CHIR), FGF2, LIF and Activin A (ActA), which have been used to maintain XEN cells *in vitro* (Park *et al.* 2021, Wu *et al.* 2023). B27 supplements were also selected based on our preliminary screening, reducing lipid droplet formation within XEN cells (Supplementary Fig. S1). Then, the optimization of culture conditions was performed with newly derived pig XEN cells that were generated from *in vitro*-produced BLs based on our previous study (Oh *et al.* 2022). Approximately 14 days after seeding BLs on the feeder, four cell types representing Epi, PrE, TE and mesoderm-like cells arose from spreading outgrowths (Fig. 1A) as previously reported (Oh *et al.* 2022). PrE cell types were separated and incubated in the culture medium containing CHIR, FGF2, LIF, ActA and B27 supplements. After several passages, they were maintained in the form of small and round colonies composed of lipid droplet-embedded epithelial cells (Fig. 1B) and thereafter named pig XEN cells. Eleven IVF-derived pig XEN (IVF-pXEN) cells were produced from 31 IVF BLs (derivation efficiency 35.5%). In addition, two parthenogenetic pig XEN cells (PG-pXEN) cells were produced from 15 parthenogenetic BLs (derivation efficiency 13.3%) and exhibited repeatability with IVF-pXEN cells, demonstrating that pig XEN cells can be reproducibly derived from diverse embryo origins.

To optimize this culture system, we identified essential signaling molecules and supplements by removing B27 supplements, CHIR, FGF2, LIF or ActA individually from the medium during maintenance. The morphology of the colonies was monitored (Fig. 1C). Conditions without B27 supplements or LIF resulted in suppressed cell proliferation, generating highly dense and small colonies. These patterns were reinforced when FGF2 was removed, and there were no colonies. Without CHIR, the boundary of the colonies was disrupted, and the cellular density within a colony was diminished. The control group showed damaged colony integrity compared to the ActA-removed conditions, suggesting that exogenous ActA inhibited the self-renewal of pig XEN cells. Next, AP staining and immunocytochemistry for the representative XEN cell marker GATA6 were conducted to verify the responsiveness to these factors (Fig. 1D and E), which supported the results of the morphological analysis. While AP staining and GATA6 expression were homogeneously positive in the ActA-withdrawal group, in the other groups, the expression was detected locally within the colonies that had been disrupted (Cont., –CHIR and –FGF groups) or aggregated (–B27 and –LIF groups). In particular, the absence of LIF elicited intense GATA6 expression only in the center of the colony, indicating spontaneous differentiation around the boundary. In summary, pig XEN cells required the WNT, FGF2 and LIF signaling pathways and B27 supplements for their maintenance, and ActA compromised colony integrity by inhibiting self-renewal.

**Figure 2:**
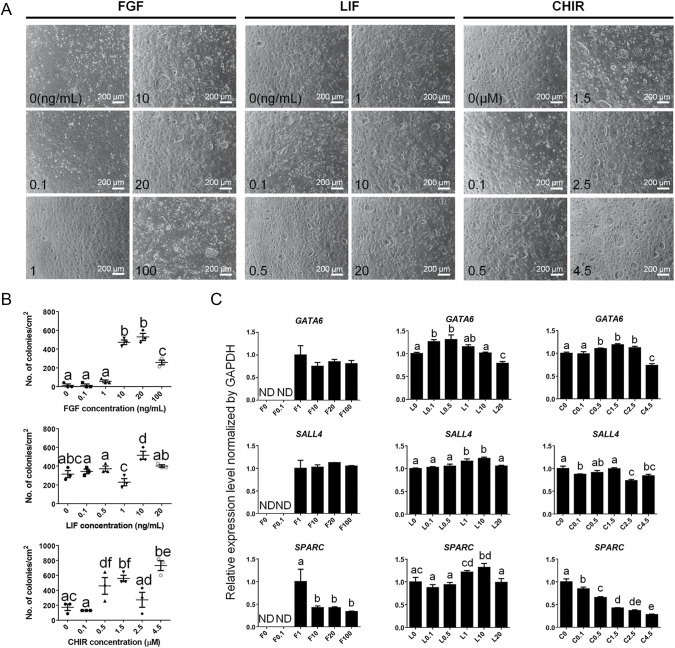
Identification of the optimal concentration of essential factors for pig XEN cells. (A) XEN cells treated with each condition. Dashed lines represent intact XEN colonies. (B) Number of XEN colonies per cm^2^. Significant differences are represented by different letters. (C) qPCR results associated with XEN (*GATA6* and *SALL4*) and differentiation markers (*SPARC*). The amount of gene expression in the F1, L0 and C0 groups is described as 1 compared to the others. F: FGF2, L: LIF, C: CHIR99021, ND: not detected. (B and C) Data are mean ± SEM. The significance of differences was determined by one-way ANOVA tests and represented by different letters, *n* = 3 technical replicates, *P* < 0.05.

In addition, to determine the optimal concentration of growth factors, including FGF2, LIF and CHIR, the colony-forming rate of XEN cells was analyzed at various concentrations (Fig. 2A and B). Low concentrations of FGF2 (under 1 ng/mL), LIF (under 1 ng/mL) and CHIR (under 0.1 μM), as well as high concentrations of FGF2 (over 100 ng/mL) and LIF (over 20 ng/mL), significantly decreased the colony-forming rate. While a high concentration of CHIR (over 4.5 μM) increased the colony-forming rate, it significantly decreased the expression of *GATA6* (Fig. 2C). Therefore, further analyses were performed under 10 ng/mL FGF2, 10 ng/mL LIF and 1.5 μM CHIR. In addition, during *in vitro* culture under O_2_ 20% conditions, the cells were detached, consistent with our previous reports that high oxygen conditions inhibited the maintenance of the pig PrE lineage *in vitro* (Oh *et al.* 2022). The reduced viability of pig XEN cells was recovered by lowering the O_2_ concentration to 5% (Supplementary Fig. S2). Consequently, subsequent analyses were conducted under 5% CO_2_ and 5% O_2_ conditions.

### Characterization of pig XEN cells

**Figure 3:**
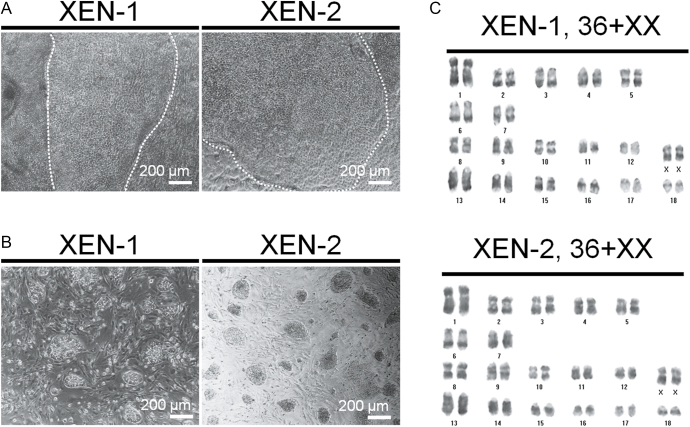
Derivation of pig XEN cells from blastocysts. (A) Derivation of XEN-1 and XEN-2 cell lines from porcine blastocysts. The images indicated 13 days after BL seeding to derive pig XEN cells. (B) Maintenance of XEN-1 and XEN-2 cell lines. The images indicated typical morphology of pig XEN cells during maintenance. (C) Karyotype of XEN-1 and XEN-2 cell lines.

**Figure 4:**
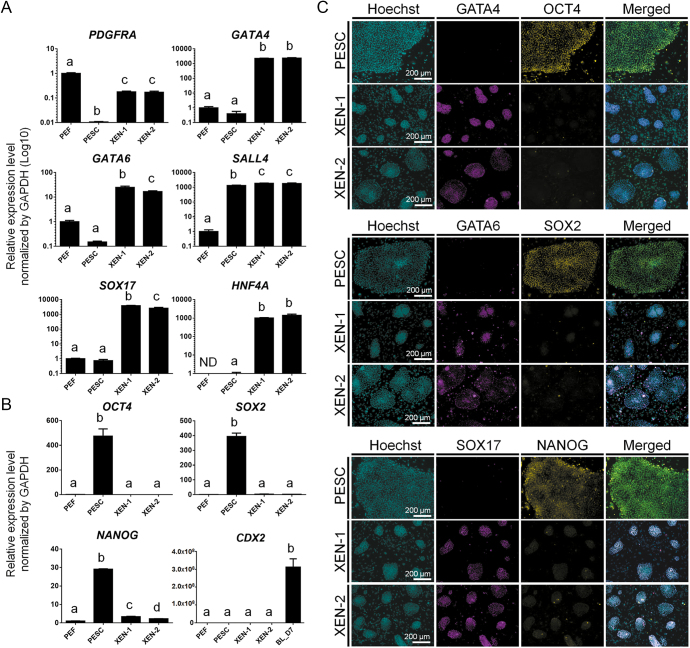
Characterization of pig XEN cells. (A and B) qPCR results for (A) XEN cell markers and (B) Epi and TE markers. The amount of gene expression in the PEF group is described as 1 compared to the others (in *HNF4A* expression, the amount in the PESC group is described as 1). ND: not detected. Data are mean ± SEM. The significance of differences was determined by one-way ANOVA tests and represented by different letters, *n* = 3 technical replicates. *P* < 0.05. PEF: porcine embryonic fibroblast, PESC: porcine embryonic stem cells, BL: blastocyst. (C) Immunostaining for XEN cell markers.

Using the above optimized culture conditions, we maintained IVF-pXEN cell lines (XEN-1 and XEN-2) with a normal karyotype (Fig. 3) and verified their XEN identity. The XEN cell lines were stably proliferated over 40 passages without losing their stemness. The ICM of BL is separated and committed to the Epi or PrE lineage, which are represented *in vitro* as ESCs and XEN cells, respectively. Accordingly, porcine ESCs (PESCs) were used with the derived XEN cell lines to compare the characteristics of the opposite ICM-derived stem cells. Using designed primers that did not detect the mouse genes to exclude expression from the mouse feeder cells, the expression of XEN-specific markers such as *PDGFRA*, *GATA4*, *GATA6*, *SALL4*, *SOX17* and *HNF4A* was investigated by qPCR (Fig. 4A). In both pig XEN cell lines, the expression levels of these genes were significantly higher than those in PESCs or porcine embryonic fibroblasts (PEFs). As previously reported, *PDGFRA* and *SALL4* are expressed not only in XEN cells but also in fibroblasts and ESCs, respectively (Lim *et al.* 2008, Donovan *et al.* 2013). In addition, our results confirmed the previous observation that pig XEN cells were negative for the Epi markers *OCT4*, *SOX2* and *NANOG* or the TE marker *CDX2* (Fig. 4B) (Park *et al.* 2021). Consistent with these results, robust expression of GATA4, GATA6 and SOX17 in both pig XEN cell lines was detected at the protein level, whereas OCT4, SOX2 and NANOG showed absent or diminished expression (Fig. 4C). These results support previous reports that showed a decrease in Epi markers concurrent with an increase in XEN markers during the establishment process from BLs toward XEN cell lines (Cho *et al.* 2012). This suggests that XEN cell lines exhibit distinct characteristics from pluripotent stem cells such as PESCs, where they have more committed stemness than pluripotency.

**Figure 5:**
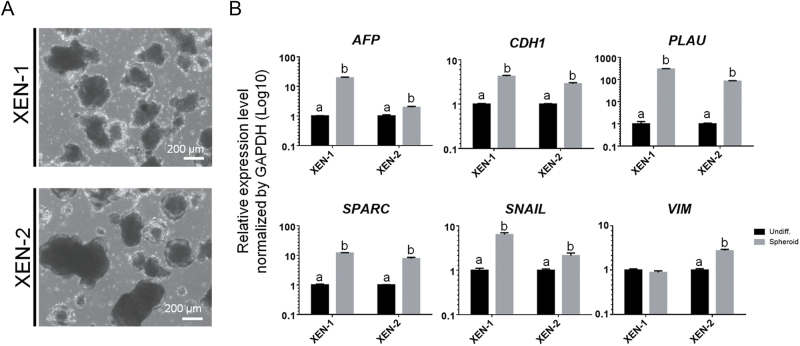
*In vitro* differentiation capacity of pig XEN cells. (A) Spheroids from XEN-1 and XEN-2 cell lines. (B) qPCR results for visceral endoderm (VE)- and parietal endoderm (PE)-associated markers in XEN-derived spheroids. The amount of gene expression in the Undiff. group is described as 1 compared to the others. Data are mean ± SEM. The significance of differences was determined by two-tailed *t*-tests and represented by different letters, *n* = 3 technical replicates, *P* < 0.05. Undiff.: undifferentiated XEN cells.

**Figure 6:**
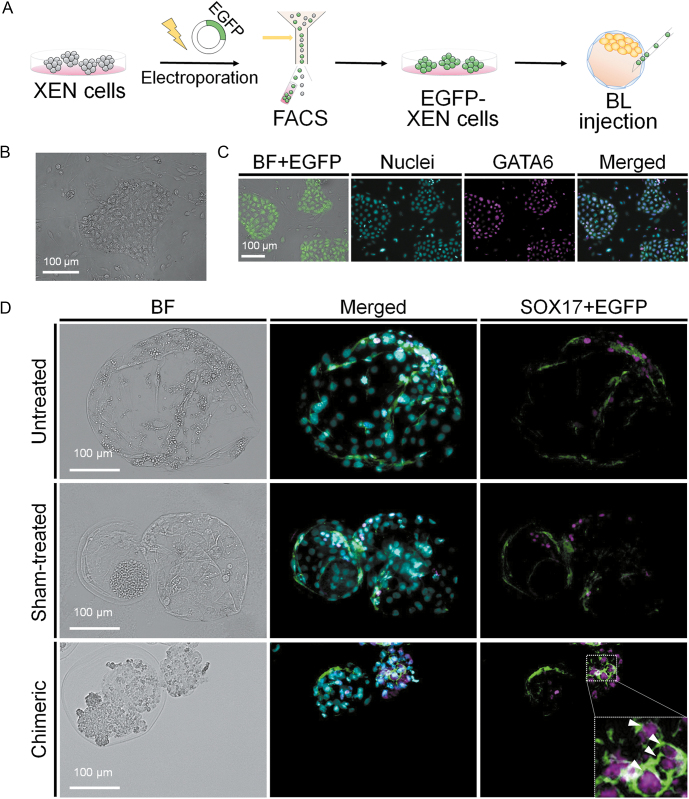
Generation of chimeric embryos with pig XEN cells in blastocysts. (A) Experimental scheme for blastocyst injection. (B) EGFP-XEN cell images. (C) Immunostaining for EGFP-XEN cells. (D) Immunostaining for D7 blastocysts injected with EGFP-XEN cells. Untreated: D7 blastocyst without injection. Sham-treated: D7 blastocyst where an injection without cells was conducted on day 5. Chimeric: D7 blastocyst where EGFP-XEN cells were injected on day 5. Nucleus (cyan), SOX17 (magenta), EGFP (green). Arrows indicate PrE lineage-contributing EGFP-XEN cells after injection.

To evaluate the differentiation ability of these XEN cell lines, we formed XEN-derived spheroids from each cell line (Fig. 5A) and induced spontaneous differentiation. Upon XEN-derived spheroid formation, their gene expression associated with parietal endoderm (PE; *AFP*, *CDH1* and *PLAU*) and visceral endoderm (VE; *SPARC*, *SNAIL* and *VIM*) was confirmed by qPCR (Fig. 5B). The expression levels in each pig XEN cell line-derived spheroid were significantly higher than those of undifferentiated (Undiff.) cell lines. It demonstrated that both cell lines possessed differentiation capabilities toward PE and VE. Furthermore, to determine whether pig XEN cells are capable of integrating into host embryonic cells and exhibiting characteristics as PrE lineages, they were injected into early embryos at day 5 after parthenogenetic activation (*n* = 139, Fig. 6A). The XEN cells were tagged by electroporation-mediated transfection of a linearized plasmid including the EGFP sequence under the EF-1α constitutive promoter. The EGFP-positive XEN cells (EGFP-XEN cells) enriched by FACS maintained self-renewal and XEN marker expression after transfection (Fig. 6B and C). Chimeric embryos with EGFP-XEN cells were generated and cultured *in vitro* for another 48 h (*n* = 88; 63.3%). Unlike untreated and sham-treated embryos, which served as negative controls, the EGFP-XEN cells incorporated into host ICM cells and co-expressed SOX17 with EGFP in the chimeric embryos (Fig. 6D). It suggests that they can participate in the early development of pre-implantation blastocysts by contributing to the PrE lineage. Taken together, we derived XEN cell lines using serum-free culture conditions, where they represented the porcine PrE lineage in terms of their molecular features and differentiation capacity.

## Discussion

In mouse and human studies, FBS-based standard ES or TS medium has been commonly employed for the derivation and proliferation of XEN cells. However, these culture conditions generate heterogeneous stem cells in terms of mixed populations of XEN cells with ES or TS cells (Cho *et al.* 2012, Niakan *et al.* 2013, Artus & Chazaud 2014). In this study, we developed a serum-free culture system for pig XEN cells with defined signaling molecules and supplements.

In the 2000s, mouse XEN cells were derived from BLs and ESCs and were characterized as reproducible and self-renewing PrE lineages *in vitro* (Fujikura *et al.* 2002, Kunath *et al.* 2005). Thereafter, mouse XEN cells have been routinely cultured in FBS-containing media supplemented with FGF4 or LIF, which have been used for TSC and ESC cultures, respectively (Niakan *et al.* 2013, Lin *et al.* 2016). However, in mice, several studies have reported that FGF receptors are absent in XEN cells and that FGF/ERK signaling is not essential for XEN cells, unlike *in vivo* PrE lineage (Kunath *et al.* 2005, Yamanaka *et al.* 2010, Cho *et al.* 2012). Rather, it has been demonstrated that the proliferation of XEN cells required LIF/STAT signaling, as exemplified by the expression of LIF receptors (Kunath *et al.* 2005, Artus *et al.* 2010, Zhong *et al.* 2018) and the effect of LIF-mediated JAK/STAT on XEN cell expansion (Morgani & Brickman 2015, Lin *et al.* 2016, Anderson *et al.* 2017). Recently, upon the efforts to establish chemically defined culture conditions for XEN cells, other signaling pathways have been identified for their maintenance. For instance, a combination of FGF4, CHIR and PDGF-AA or that of ActA, CHIR and LIF was used to derive XEN cells in mice and humans (Anderson *et al.* 2017, Linneberg-Agerholm *et al.* 2019, Ohinata *et al.* 2022, Wu *et al.* 2023). CHIR and ActA induced PrE property-exhibiting cells, and the other factors promoted their expansion. Even in pigs, although several approaches have been attempted to culture XEN cells using serum-free media, these conditions were inefficient for maintenance. KSR-based media supplemented with FGF4 showed cell senescence, and N2B27-based media containing FGF2 and LIF exhibited prolonged doubling time compared to FBS-based media (Shen *et al.* 2019, Li *et al.* 2020, Park *et al.* 2021). Taken together, these findings suggest that these culture conditions are sub-optimized for the self-renewal of pig XEN cells.

We investigated optimal conditions for pig XEN cell maintenance through supplementation of XEN cell growth-promoting factors. Ultimately, the FGF, LIF and WNT signaling pathways and B27 supplements are essential for maintaining pig XEN cells *in vitro*. Both the FGF and LIF signaling pathways were indispensable in pig XEN cells, unlike in mice. In particular, the cells showed critical responsiveness to FGF2 in our removal experiments, where their expansion was arrested and dispersed colonies were induced. Several studies have reported that exogenous FGF2 is involved in the development of bovine PrE and the proliferation of porcine XEN cells (Yang *et al.* 2011, Shen *et al.* 2019, Li *et al.* 2020, Park *et al.* 2021). Moreover, a series of experiments using FGF or its downstream inhibitors revealed the dependency of porcine XEN cells on this signaling pathway for their viability and proliferation (Zhang *et al.* 2021). In our results, the LIF-removed condition caused the failure of XEN cell proliferation and the gradual differentiation around the colony boundaries, confirming the essential role of LIF. The withdrawal of CHIR induced a low level of GATA6 expression, damaged colony integrity and weakened AP signals, demonstrating the role of the WNT signaling pathway in eliciting XEN properties through regulation of Gata6 expression, as in mice (Anderson *et al.* 2017, Ohinata *et al.* 2022). The withdrawal of FGF2, LIF or CHIR resulted in damaged cell self-renewal, indicating that all three factors are required for cell maintenance. However, no significant effect on maintenance was observed when ActA was omitted; rather, these culture conditions were preferred in terms of colony integrity. In pigs, the PrE lineage expresses abundant TGF family members on its surface, and treatment with SB431542, a TGF-β pathway inhibitor, decreases the viability of XEN cells (Zhang *et al.* 2021). This suggests that although endogenous TGF-β signaling is important for self-renewal in our pig XEN cells, additional ActA is not required because the paracrine effect of ActA secreted by the feeder cells is sufficient.

The pig XEN cells recapitulated the PrE lineages with porcine species-specific characteristics, including the involvement of lipid metabolism and NANOG/GATA co-expression. Cellular lipid droplets have been observed in porcine XEN cells, as in a previous study, mirroring the energy-enriched endodermal cells of the yolk sac for embryo development (Park *et al.* 2021). In our results, the accumulated lipid droplets and thereby poor self-renewal were alleviated by treatment with B27 supplements (Supplementary Fig. S1). These supplements contain vitamins, proteins and other components. Of these, corticosterone, which promotes lipolysis as a glucocorticoid, as well as fatty acids (linoleic/linolenic acids), seems to be responsible for improving the metabolic aspects of XEN cells by augmenting the amount of fatty acids in the cultures (Mir *et al.* 2021). According to our previous studies, fatty acids, especially linoleic acids, are involved in the development of embryos and the maintenance of ESCs in pigs (Choi *et al.* 2019, Lee *et al.* 2022). In light of these observations, the addition of B27 supplements advanced the metabolism of pig XEN cells, leading to enhanced expansion and decreased size of lipid droplets. In addition to these metabolic properties, pig XEN cells also exhibited a distinct genetic signature from other mammals due to differences in developmental programs. Floating in the uterus, porcine embryos sequentially undergo more advanced development, including second lineage segregation, PrE proliferation and migration and gastrulation, in contrast to mice and humans, whose embryos are implanted immediately after BL hatching (Perez-Gomez *et al.* 2021). Transcriptomic analysis demonstrated that the antagonism of NANOG/GATA may not be a driving force for cell fate decisions between Epi and PrE in pigs (Cao *et al.* 2014, Piliszek & Madeja 2018). In mice, *Nanog*, which is expressed from the morula stage, mutually represses *Gata6* in ICM cells from E4.5, resulting in the separation of Nanog+ Epi and Gata6+ PrE. However, in pigs, NANOG is expressed not from the morula but from ICM in early BL, and GATA6 is expressed in all three lineages of BL (Perez-Gomez *et al.* 2021). Some observations have reported co-expression of NANOG and GATA6 in early PrE cells in pigs, which is similar to our results, where mild NANOG expression was detected (Park *et al.* 2021). Altogether, our XEN cells reflect porcine species specificity in the PrE lineage, providing opportunities for discovering in-depth species-specific development in comparative developmental biology.

## Conclusion

**Figure 7:**
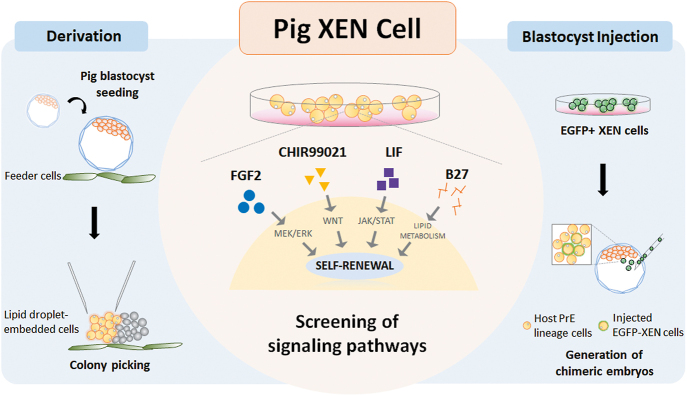
Schematic summary of results.

In this study, we derived stable pig XEN cell lines from BLs and developed a serum-free culture system optimized for their maintenance (Fig. 7). The WNT, FGF and LIF signaling pathways and B27 supplements are essential for rapid proliferation without spontaneous differentiation. As these optimized culture systems are based on serum-free medium, reproducible and homogeneous pig XEN cells can be cultured, and their self-renewal ability with rapid proliferation allows large quantities of culture. In addition, their differentiation capacity and cellular identity as a PrE lineage were confirmed by spheroid formation and chimeric embryo generation, respectively. They can be employed as a cell culture model representing extraembryonic endodermal lineages, a useful *in vitro* tool for studying mammalian development, including early lineage segregation and embryo patterning. Notably, XEN cell lines can be used to reconstruct a three-dimensional embryo model, providing a novel platform to promote the understanding of mammalian embryogenesis. Finally, our serum-free culture systems for XEN cell maintenance can shed light on more precise key molecules involved in PrE lineage development by excluding the effect of serum-derived factors.

## Supplementary materials



## Declaration of interest

The authors declare that there is no conflict of interest that could be perceived as prejudicing the impartiality of the work reported.

## Funding

This work was supported by the BK21 Four program, the National Research Foundation of Koreahttps://doi.org/10.13039/501100003725 (NRF) grant (NRF-2023R1A2C1005026; RS-2024-00408242); and the Korea Evaluation Institute of Industrial Technologyhttps://doi.org/10.13039/501100003662 (KEIT) (20012411).

## Author contribution statement

J Jeong, D-K Lee, K-H Choi, D-W Kim, S Lee, J-N Oh, Y Ahn and C-K Lee designed the research. J Jeong, D-K Lee, K-H Choi, D-W Kim, S Lee, J-N Oh and Y Ahn performed, analyzed and interpreted all experiments. J Jeong and C-K Lee wrote the paper. C-K Lee approved the final manuscript.

## References

[bib1] Anderson KGV, Hamilton WB, Roske FV, et al. 2017 Insulin fine-tunes self-renewal pathways governing naive pluripotency and extra-embryonic endoderm. Nat Cell Biol 19 1164–1177. (10.1038/ncb3617) 28945231

[bib2] Artus J & Chazaud C 2014 A close look at the mammalian blastocyst: epiblast and primitive endoderm formation. Cell Mol Life Sci 71 3327–3338. (10.1007/s00018-014-1630-3) 24794628 PMC11113690

[bib3] Artus J, Panthier JJ & Hadjantonakis AK 2010 A role for PDGF signaling in expansion of the extra-embryonic endoderm lineage of the mouse blastocyst. Development 137 3361–3372. (10.1242/dev.050864) 20826533 PMC2947752

[bib4] Cao SY, Han JY, Wu J, et al. 2014 Specific gene-regulation networks during the pre-implantation development of the pig embryo as revealed by deep sequencing. BMC Genom 15 4. (10.1186/1471-2164-15-4) PMC3925986 24383959

[bib5] Cho LT, Wamaitha SE, Tsai IJ, et al. 2012 Conversion from mouse embryonic to extra-embryonic endoderm stem cells reveals distinct differentiation capacities of pluripotent stem cell states. Development 139 2866–2877. (10.1242/dev.078519) 22791892 PMC3403099

[bib6] Choi KH, Lee DK, Kim SW, et al. 2019 Chemically defined media can maintain pig pluripotency network in vitro. Stem Cell Rep 13 221–234. (10.1016/j.stemcr.2019.05.028) PMC6626979 31257130

[bib7] Debeb BG, Galat V, Epple-Farmer J, et al. 2009 Isolation of Oct4-expressing extraembryonic endoderm precursor cell lines. PLoS One 4 e7216. (10.1371/journal.pone.0007216) 19784378 PMC2747266

[bib8] Donovan J, Shiwen X, Norman J, et al. 2013 Platelet-derived growth factor alpha and beta receptors have overlapping functional activities towards fibroblasts. Fibrogenesis Tissue Repair 6 10. (10.1186/1755-1536-6-10) 23663505 PMC3667071

[bib9] Fujikura J, Yamato E, Yonemura S, et al. 2002 Differentiation of embryonic stem cells is induced by GATA factors. Genes Dev 16 784–789. (10.1101/gad.968802) 11937486 PMC186328

[bib10] Kunath T, Arnaud D, Uy GD, et al. 2005 Imprinted X-inactivation in extra-embryonic endoderm cell lines from mouse blastocysts. Development 132 1649–1661. (10.1242/dev.01715) 15753215

[bib11] Lee DK, Choi KH, Oh JN, et al. 2022 Linoleic acid reduces apoptosis via NF-kB during the in vitro development of induced parthenogenic porcine embryos. Theriogenology 187 173–181. (10.1016/j.theriogenology.2022.05.003) 35596974

[bib12] Li Y, Wu S, Yu Y, et al. 2020 Derivation of porcine extraembryonic endoderm-like cells from blastocysts. Cell Prolif 53 e12782. (10.1111/cpr.12782) 32196806 PMC7162807

[bib13] Lim CY, Tam WL, Zhang J, et al. 2008 Sall4 regulates distinct transcription circuitries in different blastocyst-derived stem cell lineages. Cell Stem Cell 3 543–554. (10.1016/j.stem.2008.08.004) 18804426

[bib14] Lin J, Khan M, Zapiec B, et al. 2016 Efficient derivation of extraembryonic endoderm stem cell lines from mouse postimplantation embryos. Sci Rep 6 39457. (10.1038/srep39457) 27991575 PMC5171707

[bib15] Linneberg-Agerholm M, Wong YF, Romero Herrera JA, et al. 2019 Naive human pluripotent stem cells respond to Wnt, Nodal and LIF signalling to produce expandable naive extra-embryonic endoderm. Development 146 dev180620. (10.1242/dev.180620) 31740534

[bib16] Livak KJ & Schmittgen TD 2001 Analysis of relative gene expression data using real-time quantitative PCR and the 2(T)(-Delta Delta C) method. Methods 25 402–408. (10.1006/meth.2001.1262) 11846609

[bib17] Mir N, Chin SA, Riddell MC, et al. 2021 Genomic and non-genomic actions of glucocorticoids on adipose tissue lipid metabolism. Int J Mol Sci 22 8503. (10.3390/ijms22168503) 34445209 PMC8395154

[bib18] Morgani SM & Brickman JM 2015 LIF supports primitive endoderm expansion during pre-implantation development. Development 142 3488–3499. (10.1242/dev.125021) 26395492

[bib19] Niakan KK, Schrode N, Cho LT, et al. 2013 Derivation of extraembryonic endoderm stem (XEN) cells from mouse embryos and embryonic stem cells. Nat Protoc 8 1028–1041. (10.1038/nprot.2013.049) 23640167 PMC3927835

[bib20] Oh JN, Jeong J, Lee M, et al. 2022 Characterization of multitype colonies originating from porcine blastocysts produced in vitro. Front Cell Dev Biol 10 918222. (10.3389/fcell.2022.918222) 36172290 PMC9510650

[bib21] Ohinata Y, Endo TA, Sugishita H, et al. 2022 Establishment of mouse stem cells that can recapitulate the developmental potential of primitive endoderm. Science 375 574–578. (10.1126/science.aay3325) 35113719

[bib22] Park JK, Kim HS, Uh KJ, et al. 2013 Primed pluripotent cell lines derived from various embryonic origins and somatic cells in pig. PLoS One 8 e52481. (10.1371/journal.pone.0052481) 23326334 PMC3543426

[bib23] Park CH, Jeoung YH, Uh KJ, et al. 2021 Extraembryonic endoderm (XEN) cells capable of contributing to embryonic chimeras established from pig embryos. Stem Cell Rep 16 212–223. (10.1016/j.stemcr.2020.11.011) PMC7897585 33338433

[bib24] Perez-Gomez A, Gonzalez-Brusi L, Bermejo-Alvarez P, et al. 2021 Lineage differentiation markers as a proxy for embryo viability in farm ungulates. Front Vet Sci 8 680539. (10.3389/fvets.2021.680539) 34212020 PMC8239129

[bib25] Piliszek A & Madeja ZE 2018 Pre-implantation development of domestic animals. Curr Top Dev Biol 128 267–294. (10.1016/bs.ctdb.2017.11.005) 29477166

[bib26] Ralston A 2018 XEN and the art of stem cell maintenance: molecular mechanisms maintaining cell fate and self-renewal in extraembryonic endoderm stem (XEN) cell lines. Adv Anat Embryol Cell Biol 229 69–78. (10.1007/978-3-319-63187-5_6) 29177765

[bib27] Seguin CA, Draper JS, Nagy A, et al. 2008 Establishment of endoderm progenitors by SOX transcription factor expression in human embryonic stem cells. Cell Stem Cell 3 182–195. (10.1016/j.stem.2008.06.018) 18682240

[bib28] Shen QY, Yu S, Zhang Y, et al. 2019 Characterization of porcine extraembryonic endoderm cells. Cell Prolif 52 e12591. (10.1111/cpr.12591) 30896067 PMC6536407

[bib29] Wu B, Yang Z, Liu Y, et al. 2023 A chemically defined system supports two distinct types of stem cell from a single blastocyst and their self‐assembly to generate blastoid. Cell Prolif 56 e13396. (10.1111/cpr.13396) 36593753 PMC10280139

[bib30] Yamanaka Y, Lanner F & Rossant J 2010 FGF signal-dependent segregation of primitive endoderm and epiblast in the mouse blastocyst. Development 137 715–724. (10.1242/dev.043471) 20147376

[bib31] Yang QE, Fields SD, Zhang K, et al. 2011 Fibroblast growth factor 2 promotes primitive endoderm development in bovine blastocyst outgrowths. Biol Reprod 85 946–953. (10.1095/biolreprod.111.093203) 21778141

[bib32] Zhang S, Chen T, Chen N, et al. 2019 Implantation initiation of self-assembled embryo-like structures generated using three types of mouse blastocyst-derived stem cells. Nat Commun 10 496. (10.1038/s41467-019-08378-9) 30700702 PMC6353907

[bib33] Zhang ML, Jin Y, Zhao LH, et al. 2021 Derivation of porcine extra-embryonic endoderm cell lines reveals distinct signaling pathway and multipotency states. Int J Mol Sci 22 12918. (10.3390/ijms222312918) 34884722 PMC8657774

[bib34] Zhao Y, Zhao T, Guan J, et al. 2015 A XEN-like state bridges somatic cells to pluripotency during chemical reprogramming. Cell 163 1678–1691. (10.1016/j.cell.2015.11.017) 26686652

[bib35] Zhong Y, Choi T, Kim M, et al. 2018 Isolation of primitive mouse extraembryonic endoderm (pXEN) stem cell lines. Stem Cell Res 30 100–112. (10.1016/j.scr.2018.05.008) 29843002

