# Supplementary material for: Defining core signaling pathways for supporting in vitro maintenance of pig extraembryonic endoderm (XEN) cells
Source: Reproduction. 2025 Mar 11;169(4):e240393. doi: 10.1530/REP-24-0393 (PMC11906125; doi:10.1530/REP-24-0393)
Supplement: Supplementary file 1 [file supplementary_materials.pdf]

Supplementary Information

Defining core signaling pathways for supporting *in vitro* maintenance of pig extraembryonic endoderm (XEN) cells

Author Information

Jinsol Jeong[1], Dong-Kyung Lee[1,2], Kwang-Hwan Choi[1,2], Dong-Wook Kim[1], Seokjong Lee[1], Jong-Nam Oh[1], Yelim Ahn[1] and Chang-Kyu Lee[1,3*]

[1] Department of Agricultural Biotechnology, Animal Biotechnology Major, and Research Institute of Agriculture and Life Science, Seoul National University, Seoul 08826, Korea

[2] Research and Development Center, Space F corporation, Hwasung 18471, Gyeonggi-do, Korea

[3] Institute of Green Bio Science and Technology, Seoul National University, Pyeong Chang, Kangwon-do 25354, Korea

Supplementary Table S1. Primer sets used for qPCR.

Gene		Primer sequence (5' > 3')	Annealing Temperature (°C)	Product size (bp)
GAPDH	F	TGCTCCTCCCCGTTCGAC	60	100
	R	ATGCGGCCAAATCCGTTC		
OCT4	F	CTTGGAGAGCCCTGGTTTTACT	60	159
	R	GCCAGGTCCGAGGATCAAC		
SOX2	F	CGGCGGTGGCAACTCTAC	60	100
	R	TCGGGACCACACCATGAAAG		
NANOG	F	CATCTGCTGAGACCCTCGAC	60	195
	R	GGGTCTGCGAGAACACAGTT		
CDX2	F	GCCAAGTGAAAACCAGGACGA	60	120
	R	GCTCGGCCTTTCTCCGAATG		
PDGFRA	F	CAGGTTGGAGGGAGATGGAC	60	96
	R	AGTTGCGGAGGTTGGATT		
GATA4	F	CGACACCCTAATCTCGATATGTTTG	60	156
	R	CCGGCTGATGCCATTCATCT		
GATA6	F	ATCACCATCACCACCCAAGT	60	111
	R	CGCGACTCTGTAGACTGTGC		
SALL4	F	CAGGAGTACCAGAGCCGAAG	60	107
	R	ACCTCGGGAGACTTGGACTT		
SOX17	F	TGGTTGAATCTTGAGGTCTGC	60	119
	R	CAGGGTGTAGGTGTGTGATGA		
HNF4A	F	CTCAGCAACGGACAGATGTG	60	114
	R	CAGGAGCTTGTAGGGCTCAG		
AFP	F	CACCTTTCCAGGTTCCAGAA	60	106
	R	AAGGGGTGCCTTCTTGCTAT		
CDH1	F	CACCTCACGGGAATTGTCTT	60	106
	R	TTATCAGCACCCACGCAATA		
PLAU	F	AAGGGCTCTGACATTCCATG	60	95
	R	CCGGCTCTTACACTGACACA		
SPARC	F	GGACCATCAGTCCTCTGGAA	60	111
	R	AGTTCTGCGTCTCCCAAAGA		
SNAIL	F	CCCGCTGACTCTCAAGGTTC	60	109
	R	GGGCTTCTGTGGCAGTAAGG		
VIM	F	CTCCCTTTGCCCACACCC	60	144
	R	CCGAACATTCTGCGGTAGGA		

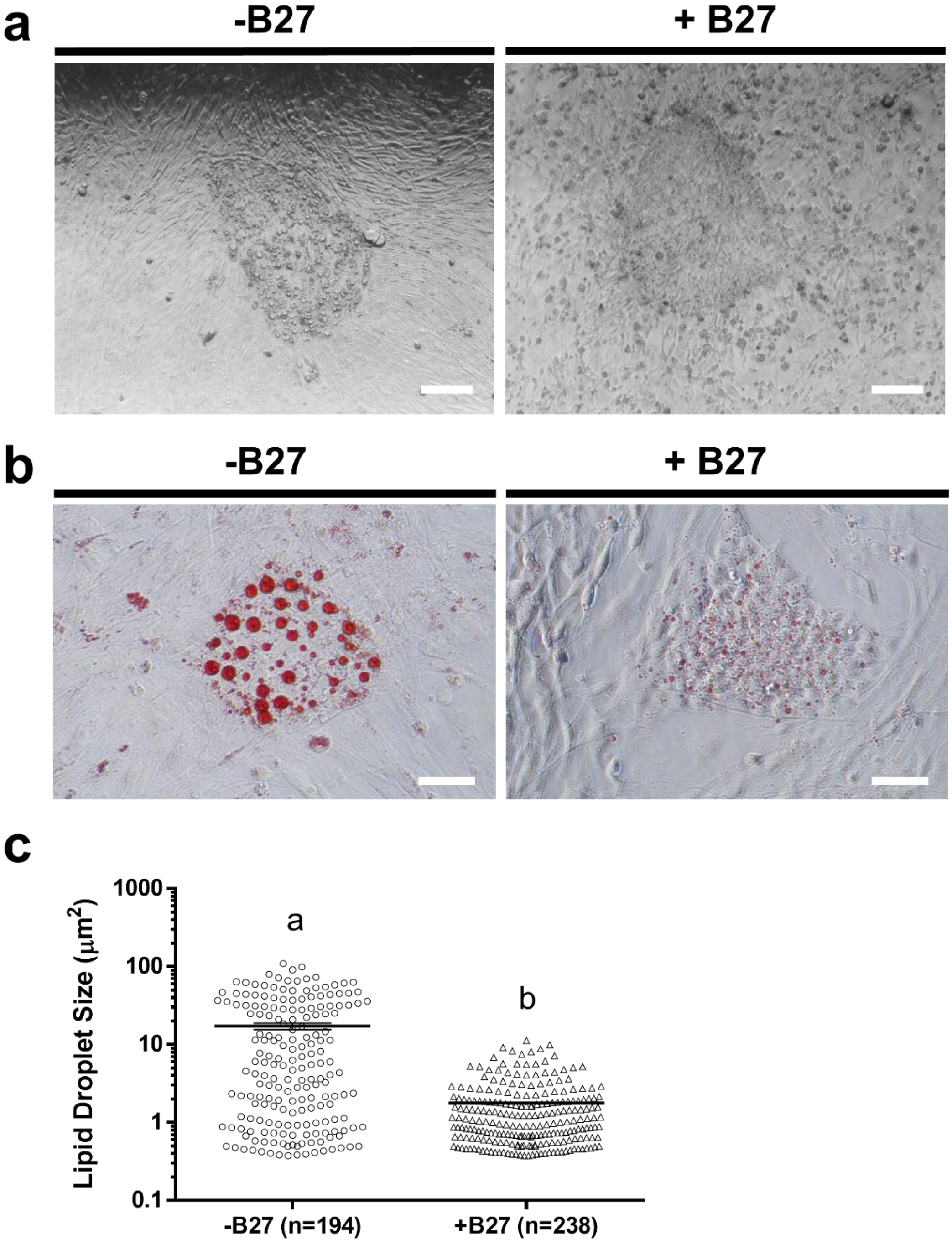

Supplementary Figure S1. The effect of B27 supplements on lipid droplets in pig XEN cells.

Changes in lipid droplets within XEN cells following B27 supplement treatment. The pig XEN cells were cultured either in medium containing B27 supplements ('+B27') or in medium without B27 supplements ('-B27'). (a) Morphology of XEN cells under each condition. Scale bar: 200 μm (b) Oil Red O staining images. Scale bar: 50 μm (c) Quantification of Oil Red O staining by measuring the area of individual lipid droplets. Data are mean± s.e.m. The significance of differences was determined by two-tailed t-tests and represented by different letters. $p < 0.05$.

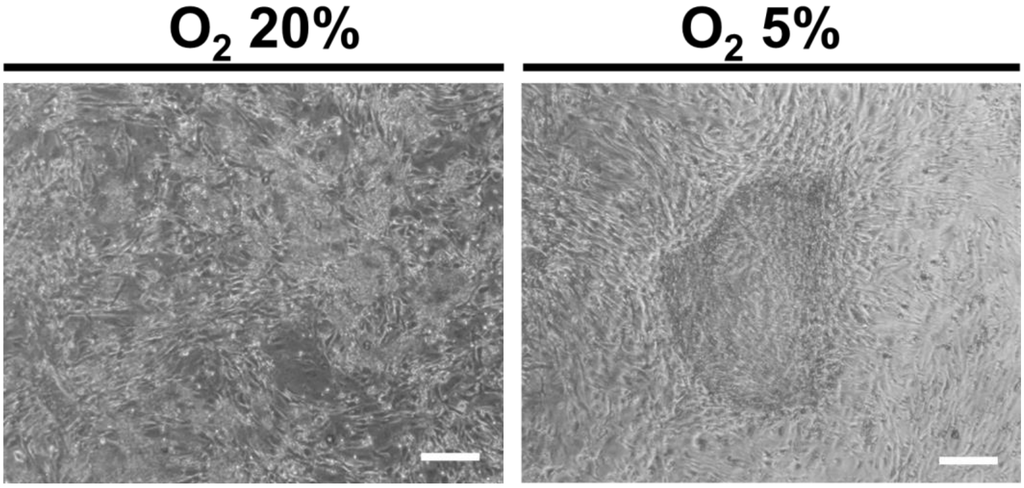

Supplementary Figure S2. Modulation of air conditions for pig XEN cell maintenance.

XEN cell morphology depending on O_2 conditions. The left and right panels indicated XEN cells cultured in O_2 20% CO_2 5% and O_2 5% CO_2 5%, respectively. Scale bar: 200 μm
